# Experiences of parents and patients hospitalised in a child psychiatric unit for anorexia nervosa after reorganisation of care imposed by the COVID-19 Pandemic in France: a qualitative study—The QUALICOVID study

**DOI:** 10.1186/s40337-022-00579-w

**Published:** 2022-04-25

**Authors:** Pauline Sourlier, Sophie Bozzi, Bernard Kabuth, Marilou Lamourette, Fabienne Ligier

**Affiliations:** 1 Service de Psychiatrie de l’Enfant et de l’Adolescent, Pôle Universitaire de Psychiatrie de l’Enfant et de l’Adolescent, Centre Psychothérapique de Nancy, 1 Rue du Docteur Archambault, 54520 Laxou, France; 2 EA 4432 PRISME, Université de Lorraine, 54000 Nancy, France; 3 EA 4360 APEMAC, Université de Lorraine, 54000 Nancy, France

**Keywords:** Anorexia nervosa, Care reorganisation, Hospitalisation, COVID-19 pandemic, Adolescent psychiatry, Qualitative study

## Abstract

**Background:**

Anorexia nervosa is a serious, albeit common mental illness that generally occurs during adolescence. Although outpatient care is recommended, hospitalisation is sometimes required. There is a dedicated hospitalisation unit caring for children and adolescents presenting with anorexia nervosa in Nancy, France. However, on 16 March 2020, a national lockdown was declared by the French government as the COVID-19 pandemic escalated in France. This resulted in the adjustment of hospital admissions accompanied by premature discharge and an intensive outpatient care programme. In the light of such changes, consideration should be given to the potential impact of changes in the care pattern for anorexic patients and their parents. The purpose of our study was to explore the experiences of anorexia nervosa patients hospitalised in the unit, and their parents, following changes in the care strategy.

**Methods:**

The study was conducted between weeks four and eight after lockdown was announced. The study cohort included all the patients treated for anorexia nervosa and hospitalised in the treatment unit before 16 March 2020 and their parents. A qualitative method was used and every subject was offered a semi-structured interview. Data were analysed by means of inductive thematic analysis.

**Results:**

Seven superordinate themes were identified: positive aspects, concerns, preparation, loss of landmarks and hospital security, gradual return to a “normal” life, relational aspects and the likelihood of disease progression. Moreover, all the parents and patients were satisfied with the intensive outpatient care offered on discharge.

**Conclusion:**

Despite initial ambivalence, all patients and their parents viewed this unexpected hospital discharge positively in these exceptional conditions. This suggests that restructuring the care programme could prove beneficial with increasing use of outpatient management, thereby reducing the length of the hospital stay and adjusting the return to school.

*Trial registration*: ID-RCB 2020-A01101-38—This project was approved by the *Comité de Protection des Personnes (CPP) Sud Méditerranée IV [South Mediterranean IV Ethics Committee (EC)]* on 5 May 2020.

## Plain English summary

Anorexia nervosa is a common mental health condition in the adolescent population. A set care protocol is in place and patients presenting this condition experience difficulty in adapting to change. In 2020, the COVID-19 pandemic was escalating in France and a national lockdown was declared by the French government on 16 March. In Nancy, France, we opted for premature discharge where possible and an intensive outpatient care programme in accordance with government recommendations. The purpose of our study was to investigate the experiences of patients and their parents regarding changes in the care strategy following lockdown. We found that patients and parents viewed the reorganisation of care in a positive light, particularly in terms of absence from school and intensive outpatient care. Our research could also benefit adolescents presenting with anorexia nervosa and undergoing restructuring of their care programme.

## Background

Anorexia nervosa is a serious mental illness defined by the DSM-V [1] as a restriction of energy intake relative to requirements, an intense fear of gaining weight or becoming overweight and a disturbance in one’s body perception, leading to a significantly low body weight in relation to their age, sex, developmental trajectory, and physical health (less than minimally normal/expected).

A lifetime prevalence of up to 2.2% has been recorded in western women [2, 3]. This problem generally occurs during adolescence [4] with two peaks at around 14 and 18 years of age and occurring on average age at 17 years of age [5]. The consequences of anorexia nervosa can be serious and the mortality rate is one of the highest with regard to mental illnesses [6, 7]. Indeed, compared to a non-clinical population, the premature death rate is approximately 5.2 [8] to 6.2 [9] times higher. This excessive mortality rate is due to the somatic complications of starvation and suicide.

Psychiatric care is therefore a crucial issue. In France, the Haute Autorité de Santé (HAS) [French National Authority for Health] produced clinical practice guidelines for the treatment of anorexia nervosa [10] in 2010. Multidisciplinary outpatient care is preferred and is also supported by many other international organisations [11] such as the American Psychiatric Association (APA) [12] and the National Institute for Health and Care Excellence (NICE) [13]. The HAS guidelines cite medical (BMI, refusal to eat or drink, bradycardia or tachycardia, paraclinical criteria, etc.), psychiatric (suicide attempt, specific suicide plan, related psychiatric disorder sufficiently severe to warrant hospitalisation, etc.), behavioural (inability to control obsessive thoughts, etc.) and environmental (severe family conflict, no outpatient treatment possible due to lack of facilities, etc.) criteria for full hospital admission [10]. The latter is mostly governed by the combination and progression of these criteria and often indicates a serious stage in the disease. Hospitalisation should last “for as long as necessary” [10] and “the patient’s weight should be stabilised before discharge at the level reached during hospitalisation, in order to reduce the risk of relapse” [10]. No specific criteria other than weight are specified contrary to NICE recommendations [13], which state that people should not be discharged “solely because they have reached a healthy weight”. Regardless of treatment strategy, HAS guidelines [10] also recommend that care be provided by at least two practitioners—a psychiatrist and a physician with regular exchanges among the different professionals involved. The recovery of an appropriate weight is the treatment objective but this can often take several months to achieve.

Within the Pôle Universitaire de Psychiatrie de l'Enfant et de l'Adolescent du Centre Psychothérapique de Nancy (France), children and adolescents with anorexia nervosa are treated in a hospitalisation unit based on the criteria and recommendations defined by the HAS. A paediatrician defines the target weight corresponding to the 25^th^ BMI percentile for the patient’s age. In the event of hospitalisation, a therapeutic contract with different intermediate weights is agreed between the care team, parents and the patient. The care contract sometimes provides a period of therapeutic separation depending on the patient’s age and various individual criteria. It allows patients to work on individualisation and empowerment. However*,* the role of the family remains crucial and must be integrated into the care package [10]. In the unit, consultations are regularly held with parents and the psychoeducation team. The effectiveness of familial therapy has already been broadly demonstrated [14, 15].

Anorexia nervosa is a chronic disease evolving over several years [16, 17], thereby justifying continuity of care from hospital discharge onwards. Consequently, “it is recommended that the coordination of care should be backed up by regular exchanges among the different professionals involved” [10] to ensure the best possible link between outpatient and inpatient care. Shortly before the target weight is reached, home leave is organised to allow a gradual return to “normal” life (half a day at first followed by two or even three days). When patients return home, outpatient care is prepared by the health care team, the patient and their family. Previous outpatient care can continue or a new follow-up strategy is set up. Hospital discharge is approved when patients have reached their target weight and maintain it for at least 2 days (for one week in the case of a second hospitalisation).

During the first quarter of 2020, France was progressively affected by the COVID-19 pandemic. A nationwide lockdown was declared and new adjustments regarding hospital admissions were introduced by the French Government on 16 March 2020 [18]. Indeed, the recommendations provided that: “Hospitalisation should be ended if it is possible to organise outpatient care. Patients for whom outpatient care is feasible should return home with close, adapted post-hospitalisation monitoring in order to guarantee continuity of care”. The parents of patients whose clinical condition was compatible with discharge were offered to prematurely end their period of hospitalisation and intensive outpatient care was instead made available. In the pandemic context, continuity of care could not be ensured from the time of hospital discharge because everything was carried out at pace and due to staff shortages in the outpatient facilities. Outpatient care was therefore delivered by the hospital team. Weekly outpatient consultations were maintained—particularly for weight monitoring purposes—coupled with telephone interviews, teleconsultations and a continuous telephone hotline provided by the health care team. If hospitalisation could not be ended because of somatic, psychiatric or familial criteria, it continued with adjustments: meals were taken alone for 4 weeks (thereafter, meals were taken with other patients but in compliance with the 2-m social distancing guidelines). Therapeutic group activities were stopped. Only individual activities were organised. Patients could not leave the unit to walk freely around the hospital. Visits were only permitted under exceptional circumstances (even if the care contract provided for therapeutic separation) but only one parent could attend. Visits were organised in a special room outside the unit and medical interviews with the patient’s family were made available via videoconferencing links. Otherwise, patients could have interviews with nursing staff on demand, as per usual, and medical consultations continued at the same pace (at least two individual medical consultations and one family teleconsultation per week).

Given these exceptional circumstances, we considered the potential impact of changes in the pattern of care for anorexic patients and their parents.

This led us to conduct a qualitative investigation into the experiences of anorexia nervosa patients hospitalised in a paediatric and adolescent psychiatric ward in Nancy, France, and their parents, regarding changes in care programmes in the context of the COVID-19 health crisis. Our study was conducted between the fourth and eighth week of lockdown (four weeks allowed patients to readjust to life at home and a period of eight weeks is often commensurate with disease recurrence following rapid weight gain).

## Methods

### Study population

The study population included all anorexia nervosa patients who were hospitalised in the care unit on the day that the first nationwide lockdown was declared, i.e., before 16 March 2020. Their parents were also included. At that time, only female patients were hospitalised. No new subject was enrolled in the study after this date, even if a new anorexia nervosa patient was admitted to the unit.

#### Inclusion criteria

For children

- Hospitalised in the care unit on 16 March 2020.
- Diagnosed with anorexia nervosa (ICD10 F50.0).
- Fluent French speakers.
- Having obtained written parental consent.

For parents

- The child satisfies the above-mentioned inclusion criteria.
- Fluent French speakers.
- Having been informed of the study, having received the relevant information and having no objection to participating in the study.

#### Non-inclusion criteria

- Objection to participation.

#### Inclusion modalities

Eligible patients were invited to participate the week before the semi-structured study interview. ML presented the details of the study to the patients and their parents during a standard scheduled interview. They were given a written consent form to sign, confirming their agreement to participate in the study. The written consent of both parents/guardians was obtained.

Consenting parents could be enrolled in the study even if the child refused to participate. Similarly, a consenting child could be enrolled in the study even if the parents refused to participate provided that the latter agreed to their child’s participation.

### Data collection

#### Socio-demographic and anthropometric data

Socio-demographic and anthropometric data were collected from every participant’s medical record using a collection grid specially designed for the study and including:

- The patient’s age.
- Family structure.
- The type of anorexia nervosa (restricting type or binge-eating/purging type).
- The prepubescent or pubescent form.
- The age of the patient at the onset of the disorder.
- Possible history of hospital admissions.
- Potential co-morbidities.

These data were collected to gain a better understanding of sample characteristics and to serve for comparisons.

Other data concerning hospitalisation and care were also collected:

- Weight, height and Body Mass Index (BMI) on admission to hospital, on the day lockdown was announced and one month and two months after the start of lockdown measures.
- The weight contract, if any.
- Possible rehospitalisation after lockdown.
- The monitoring procedures set up.

#### Semi-structured interview

Every participant in the study was offered a semi-structured interview—in French—conducted by the same person (FL). Interviews were carried out during the weekly follow-up consultation. The child was interviewed initially followed by the accompanying parent (while the child attended the consultation). Only one parent could accompany the child, due to sanitary conditions. The other parent was offered a telephone interview in the same week.

Each interview began with the same sentence: “How did you experience the change in your care (child)/your daughter’s care (parents) in relation to the Coronavirus crisis?” There was also time for free discussion afterwards, depending on the topics broached by the participant. Several questions were then asked to stimulate the discussion.

Patients discharged from hospital were typically asked the following questions:

- “What has changed regarding your care (child)/your daughter’s care (parents)?”
- “Did you have any concerns about this change? If so, what were they?”
- “Were you afraid that these changes would jeopardise the care?”
- “Do you think that returning home in a lockdown context is easier than under normal circumstances?”
- “How do you deal with your (child)/her (parents) weight contract?”

Patient who remained in hospital was asked the following questions:

- “How do you feel about staying in hospital?”
- “What do you think about your care now that barrier measures have been set up and hospital care has been restructured?”
- “How are you finding your meals at the moment (patient)?”
- “How do you spend time with your parents (patient)/daughter (parents)?”XX

Experiences regarding care frequency and approach, and the likelihood of readmission for children discharged from hospital were also discussed.

The interview closed with the following question: “In retrospect, do you think that these care changes have impacted the course of your illness (patient)/your daughter’s illness (parents)?”

### Data analysis

All the interviews were audio-recorded, pseudonymised and transcribed verbatim in French by FL. The data were analysed by inductive thematic analysis [19]. The authors chose this method because it produced reliable and valid findings through a straightforward and systematic set of procedures [19]. The themes are strongly linked to the data because they emerged from the data per se without trying to match the data to a pre-existing theory or framework (even if the authors cannot ignore their theoretical knowledge).

PS and SB read the interview transcripts several times to familiarise themselves with the data.

The data were then analysed to identify and define different themes. This analysis was conducted separately to maximise the validity of the results.

Finally, SB, PS, ML and FL discussed their preliminary results. Connections between themes were established. Findings were clustered into superordinate themes according to main data categories.

Considering the small sample size, only a descriptive analysis was conducted.

We chose to separate the thematic analysis of discharged patients’ (and parents’) interviews from those of patients who remained in hospital.

Selected extracts of the interviews were translated into English in order to illustrate the following themes. We endeavoured to present the original meaning as closely as possible.

### Ethical considerations

The research was performed in accordance with the Declaration of Helsinki. This project was approved by the *Comité de Protection des Personnes (CPP) Sud Méditerranée IV [South Mediterranean IV Ethics Committee (EC)]* on 5 May 2020 – ID-RCB 2020-A01101-38.

## Results

### Study population

Six patients and their parents were invited to take part in the study. They all agreed to participate, resulting in a total of eighteen semi-structured interviews.

Four of the six patients went home on the day the lockdown measures were announced. One patient went home three days later once her parents were available (patient 3). The last child remained in hospital due to a clinical condition incompatible with discharge (patient 4).

The participants’ sociodemographic and medical data are presented in Table 1. Changes in the patients’ BMI are shown in Table 2.

**Table 1:** Sociodemographic and medical data

	Age on 16 March 2020 (in years)	Age at the onset of the disorder (in years)	Duration of current hospital stay (on 16 March 2020) (in months)	Number of previous hospitalisations for AN	Comorbidities	Parental situation	Sibling ranking
P1	13	12	3	0	Depressive affects PTSD	Living together	1/2
P2	18	17	2	0	MDD	Living together	1/2
P3	17	16	2	0	MDD	Living together	2/2
P4	16	14	1	1	MDD	Living together	1/2
P5	13	10	6	3	MDD Anxiety disorder	Divorced with joint custody	2/2
P6	10	9	4	1	MDD Anxiety disorder	Living together	2/3

*AN* anorexia nervosa, *PTSD* post-traumatic stress disorder, *MDD* major depressive disorder

**Table 2:** Changes in BMI

	Target BMI	BMI on hospital admission	BMI on 16 March 2020	After 1 month	After 2 months
P1	17.8	16.0	17.2	17.5	17.6
P2	19.1	16.5	18.3	18.3	18.2
P3	NA	18.9	19.3	NA	NA
P4	18.2	13.7	14.4	14.5	14.8
P5	16.4	13.3	15.7	15.8	15.9
P6	18.9	13.2	14.5	14.7	15.0

*BMI* body mass index; *NA* not applicable

All the patients presented restrictive anorexia nervosa at the time of inclusion.

Only one patient (patient 6) was readmitted to hospital a few weeks after lockdown.

One of the patients (patient 3) was not routinely weighed during the weekly follow-up interviews because the weighing procedure caused her too much anxiety. Moreover, the BMI was within normal limits. She continued to gain weight and had no care contract.

Every patient who returned home was given a medical and nursing telephone interview the day after discharge in the presence of at least one of the parents. Another nursing telephone interview was held with the patient alone on Day 2 or Day 3. Thereafter, regular (at least bi-weekly) nursing telephone interviews were held for the first fifteen days.

For one month and a half, patients were interviewed twice a week via a face-to-face medical consultation in conjunction with weighing in the unit and a medical teleconsultation (or telephone consultation). Follow-up then took place on a weekly basis. The health care team were always available to families and patients in case support was needed. Three patients called the department during the first ten days after discharge. Sometimes they did it spontaneously and sometimes after their mother had called, as they were unable to call independently. All the mothers called the department once, generally regarding administrative issues, but sometimes because their daughter could not call unaided.

The aims were to support the patients and their families through this extremely difficult period and to continue psychotherapy, which began during hospitalisation. The psychotherapy was individual and patient-adapted.

Patients had been hospitalised for several weeks or even months. They had to gain 2 or 3 kg to achieve their target weight. Hence their weight gain was already well underway. They had been given advice on nutrition and had already worked on body recovery acceptance in the unit. The weekly face-to-face medical consultation facilitated weight monitoring to ensure that the patients were not losing weight and kept gaining weight. Moreover, every contact between the team and the patients allowed the latter to talk about their difficulties and anxieties regarding weight gain or to discuss any other issues of concern.

With regard to the patient who remained in hospital, medical and nursing interviews continued at their usual frequency: at least two individual medical consultations and one family teleconsultation per week, backed up by interviews with the care team, if necessary.

All patients continued to gain weight during the 2 months spent at home. Nevertheless, one patient (Patient 6) was readmitted 2 months after lockdown restrictions had been lifted (i.e. after 4 months) due to the recurrence of anorexic symptoms and weight loss.

### Thematic analysis

Seven superordinate themes were identified in the interviews with the discharged children and their parents. The following topics were covered: positive aspects of a premature return home, concerns, preparation, reference points and safety in hospital, gradual return to a “normal” life following lockdown, likelihood of disease progression and relational aspects.

#### Positive aspects of a premature return home

The patients had spent several months in hospital and should have stayed longer according to their weight contract. All participants, both children and parents, immediately expressed positive views about going home early:

“All I wanted to do was to get out, so I was happy straightaway.” (Patient 1)

“Immediately, I was happy and relieved that I could see my daughter again.” (Father 2)

“It was pretty good news actually, (…), so I was happy she was coming home.” (Mother 5)

Reassurance and relief were also echoed in interviews with three of the mothers. However, neither the fathers nor the children expressed these feelings.

“I was relieved at first.” (Mother 2)

“I’d rather have her with me than… than knowing she was in hospital.” (Mother 5)

“Relief that she wouldn’t be contaminated in hospital.” (Mother 6)

This positive aspect seemed to permeate through discussions of everyday life since leaving the centre for all participants:

“For me and for the moment, I’m quite happy with how it went, overall it’s going well, and it went well, so I’m basically happy.” (Patient 1)

“We think a lot of progress has been made. Well, it’s a blessing in disguise.” (Father 3)

“It's gone rather well.” (Mother 6)

#### Concerns

In an ambivalent way, faced with the immediate prospect of returning home, most of the parents expressed concerns and anxieties about a resurgence of symptoms that led to hospitalisation in the first place and thus a possible relapse:

“Let’s say we were a little apprehensive about what was going to happen.” (Father 1)

“Concerning indeed.” (Father 2)

“I was afraid to see how things would go at home.” (Mother 3)

Parents of patients with a history of hospitalisation were also concerned:

“Of course, we’re always very worried every time she comes out of the hospital as we’re always afraid she'll have a relapse.” (Father 5)

“I was worried initially. I said to myself, well I hope she won’t relapse straightaway.” (Father 5)

“When we were called, I was quite worried actually…” (Mother 6)

“The first few days, we were so worried, thinking she might well have a relapse.” (Father 6)

Only mother 5 said she didn’t feel these concerns: “*I didn’t have time to think about it and now I’ve decided not to think about it anymore othrwise*…”.

Only the three adolescents with no history of hospitalisation, who are also the oldest participants (patients 1, 2 and 3) voiced their apprehension:

“I was afraid it would happen again.” (Patient 1)

“I was panicking.” (Patient 2)

“Returning home hadn’t gone too well in the past, so I was a bit scared.” (Patient 3)

#### Preparation

Usually, the return home and the outpatient handover are prepared before the patient is finally discharged. Patients prepare for this by spending increasing periods at home ranging from half a day initially to two or sometimes even three days. This was not possible on this occasion because of the exceptional circumstances. Many of the participants (parents and children) mentioned this idea of preparation. They mostly felt insufficiently prepared, even for a patient with a history of hospitalisation (Patient 6):

“I didn’t feel ready at all.” (Patient 3)

“We weren’t really prepared; it was done in a hurry.” (Father 3)

“There was an element of panic.” (Father 3)

“There was no preparation for returning home under these circumstances.” (Mother 5)

“We didn’t really know what to expect.” (Mother 6)

Only one patient (Patient 5) said: “*I felt ready, finally ready, that’s it. I really wanted to, so I felt ready, but* …".

#### Loss of landmarks and hospital security

Apart from one mother who, when speaking about her daughter, commented that “*being in her cocoon has a reassuring element that (…) has helped her progress*” (Mother 6), all the children and parents mentioned the reassuring landmarks of the hospital setting (daily weighing, meal portions and a care team constantly on hand to support the patients):

“Getting used to the new way of working was very hard but now I think it’s going a bit better.” (Patient 2) (with reference to weighing)

“Well… there were the meals that were, I mean, on trays. The portions of the meals were well defined.” (Patient 2)

“She was under immense pressure for the first two weeks. She had such a lot of work to do, a lack of reference points because she had held on tightly, I had seen that there were a lot of rituals. It’s silly but I immediately asked her ‘what foods did you find reassuring at the hospital?’” (Mother 2).

“It’s a bit more complicated here and we’re not carers either (…). She was used to the care team, to the meals, to other people being with her. That’s for sure…” (Father 2)

“I’m not reassured at all at home. Here, I must have a medical environment.” (Patient 3)

“Without… without having any reference points from here (…) I was being weighed every morning, in my underwear, and on an empty stomach, and all that… the strictness (…) I had my bearings and now I don’t know.” (Patient 5).

“She was safe [in the hospital].” (Father 6)

“At the hospital, the, the meals, it’s quite safe because there are trays, because there are portions.” (Father 6)

#### Gradual return to a “normal” life

##### Education

All the children and their parents emphasised that lockdown made it easier to return to school as it was gradual and particularly because of the importance attached to how the patients are perceived by others.

“I was very afraid to go back to school because of how other people would view me.” (Patient 2)

“I found that when she came out of hospital, I’m talking about the previous times, (…) I thought the transition was a bit sudden, you know, with her coming home and having to return to school and then having to cope with everything.” (Mother 5).

“I think that being in her cocoon without any social interaction, without having to deal with how other people perceive her, uh… other than her close family, I think that really suits her very well. And from this perspective, lockdown restrictions will also be lifted bit by bit and a gradual return to school will also help her, as far as I’m concerned.” (Mother 6).

“She was very worried about seeing everyone at school.” (Father 6)

Furthermore, they all insisted on the fact that all pupils will go back to school at the same time which will make the patients’ return to class easier compared to the normal return to school during the academic year, which is the case following a conventional discharge.

“If I had gone home like that in the middle of the year, I think I would have felt like, ‘what happened?’ which I didn’t want at all.” (Patient 1)

“She won’t be singled out as much.” (Father 3)

“She will be less, I mean… apart from the others, she will go back at the same time as everyone else, I’m not saying it will be that simple but it may be a little easier than usual.” (Mother 5).

Only one mother (Mother 6) noted that it will be possible to avoid meals in the cafeteria thanks to this gradual return to school: “*There won’t be meals in the cafeteria straightaway and I think that must also be reassuring for her somehow*.”

##### Family/friendship

Some parents, both fathers and mothers, were reassured by the fact that they were at home all day long to be with their daughter if she needed them. This fact also helped to reassure their daughter:

“It worked out well because we were, you know… we were at home due to the lockdown, etc..” (Father 1)

“We were there and available—she wasn’t alone.” (Mother 2)

“I was also reassured as I was working remotely mean, I was at home.” (Mother 3)

“We’re there to talk about it. We can pick up on the first sign of any drop in form and can talk about it.” (Mother 6)

“The fact that we are there with her, as a family etc. is very positive.” (Father 6)

The children did not refer to this aspect.

However, one mother (Mother 2) said she was exhausted from being continually exposed to her daughter’s anorexic behaviour because of lockdown measures: “*Now I’m working from home again, thinking I can’t watch her anymore because when I spot little things, it affects me, it almost frightens me.*”

Only one parent (Father 3) commented that they cannot have meals outside with friends because of lockdown measures which was easier for his daughter: “*What’s good is that for now, we don’t have people at home and we don’t go to people’s homes, so meals are less complicated (…) We do it in steps.*”

#### Relational aspects

Only the parents mentioned relational aspects. All of them described their daughter as self-centred or at least having been so:

“There is that one thing though, the fact that she’s withdrawn into her own little world.” (Father 1)

“She’s still very self-centred. […] It’s all about her.” (Mother 2)

“She’s been very relaxed over the past week (…) whereas she wasn’t at all before, she was self-centred.” (Mother 3)

“She was very, she was very centred, very self-centred, very self-centred, talking about nothing else if you like (…) and all of a sudden, I feel that she has opened up.” (Father 5).

Some parents also mentioned the influence illness can have on the whole family not only at mealtimes but also outside:

“I’m going crazy (…) At times I feel like I can’t take it anymore … (…) it’s boring, really.” (Mother 1)

“Her sister says I don’t exist anymore, she keeps everything to herself, that’s what’s complicated.” (Mother 2)

“I feel depressed, weary, I’m fed up.” (Mother 2)

“Sometimes my husband breaks down too.” (Mother 2)

“She’s extremely hard to live with (…) She’s very hard to deal with.” (Father 5)

Two parents pointed out that their daughters were now able to hear some remarks about food without getting angry, which was not the case prior to hospitalisation:

“She’s more flexible too.” (Father 1)

“She gets it.” (Father 1)

“She’s much more cooperative now, meaning she felt that before it was doctors and parents against her, they wanted to make her fat. Now it’s doctors and parents with her and well, she understands that she needs it.” (Father 6).

#### Likelihood of disease progression

##### A better understanding of the disease

Parents and children had a better understanding of the symptoms and most of them emphasised the improvement in their relationships as a result:

“Well, when I went out, my parents were much more understanding. (…) It was much better and actually it helped me a lot to have my parents with me.” (Patient 1)

“I’d say that the difference for me is that now she knows she's anorexic so, that’s one thing, she knows she has to work at it, that it’s going to be hard, that we have to move on, etc.” (Father 1).

“Well, I realise that I used to talk a lot about weight in the morning … but now, I don’t do it anymore. I do it sometimes when I feel it’s time to put things right…” (Mother 5)

“I feel more reassured by my parents.” (Patient 6)

##### Motivation

Some parents and only one patient mentioned the concept of motivation and willingness needed to help patients recover from anorexia nervosa, thus implying that it is a constant struggle:

“At the beginning, I felt she was very determined.” (Mother 1)

“To her, coming back home was … She wanted to come back home, etc. So, I think that, to us, it also implied that she was going to make an effort (…) otherwise she would go back into hospital straightaway. So it wasn’t, I mean, we imagined that she was going to do what she had to…” (Father 1).

“I want to get out of all of this, I’m also trying not to get into it again and to be able to …" (Patient 3)

“I have the impression that it’s a challenge, the obligation to reach the discharge weight, (…) and then that gives her more motivation.” (Father 5)

##### Relationship with food

Patients expressed a persistent fear of food:

“I feared I would eat like I did before when my appetite came back.” (Patient 1)

“It scares me less; I prefer to eat something that scares me less.” (Patient 2) comparing a yoghurt and a chocolate bar.

“Nevertheless, there are always difficulties with what they eat, and I’m always comparing myself.” (Patient 3)

“- What was scary out there?—Well, that my parents fed me too much.” (Patient 6)

It was different for patient 5 who became a vegetarian: “*I eat differently. I became more attached to animals when I was discharged, I mean, even more so than before. I already knew it was going to be like that when I was in hospital and so I don’t eat animal produce anymore*”. She can eat “*with less anxiety*”.

Three parents re-established weight or dietary-related goals at home:

“At the beginning we made a list of what … (…) what scared her, every, every week we gave her something that scared her, something sweet, something savoury.” (Mother 1)

“With my husband we said we were going to set weights (…). So, we try to experiment with it, to create some sort of structure, anyway.” (Mother 2)

“We tried to tackle it in stages.” (Father 2)

“When we saw problems arising, we set up contracts with her actually.” (Mother 6)

#### Regarding the patient who remained in hospital

The main theme emerging from the interview with the patient who stayed in hospital and from the interviews with both parents was loneliness:

“Suddenly, I was all alone.” (Patient)

“It was especially boring for her, extremely boring.” (Mother)

She felt particularly lonely at mealtimes as she had to eat alone in her room. The patient and her parents said that had negative consequences:

“At the beginning, it was very complicated because we ate alone in our rooms and that didn’t help me much.” (Patient)

“I was all alone so I often hid food.” (Patient)

“It was a disaster when she ate alone in her room.” (Mother)

“I’d say that the most negative thing was when she ate alone in her room, I think.” (Father)

There was also the idea of the hospital being safe. This concept was mentioned by the patient herself: “*They think that I’m safer here than anywhere else*” and indirectly by her mother: “*She knows that I think she needs it, that there is no choice*”.

Unlike the other mothers, this mother didn’t refer to her daughter being self-centred: “*When we can be together, it goes well. (…) She tries to show an interest in how things are going at home, in what we do.”*

### Global assessment by the participants

All the parents said they were satisfied with the care offered on discharge. They emphasised the fact that they “*didn’t feel any cut off*” (Father 1), they “*could ask the care team immediately*” (Mother 2), they “*didn’t feel abandoned*” (Father 2) and they “*weren’t left to their own devices*” (Mother 5 and Father 6). This was deemed to be “*reassuring*”, “*comforting*”, depending on the terms used by the parents and children.

One patient felt that a weekly weighing session was not enough and that she would like to be weighed more often, explaining that she “*can’t check every day if (she) has put on too much weight*” (Patient 2).

Patient 5 said she felt anxious about returning to the hospital for face-to-face consultations.

Only one girl considered the possibility of being hospitalised again at the end of lockdown. The other patients do not refer to this, despite the fact that none of them had reached their discharge weight at the time of the interview.

The parents’ comments were more tempered but they did not consider rehospitalisation either, as they noted a clinical improvement in their child. However, all the parents would be willing to accept hospital readmission if recommended by a doctor.

The mother of the patient who remained in hospital expressed a desire for more frequent teleconsultations. However, she stressed that the care remained “*along the lines of what had been done before.*” As far as the father was concerned, there was a “*break*” in care.

Only Father 2 would have liked “*the process to go all the way*” because “*it was more complicated (for his daughter) to achieve the objectives that were set by the weight contract*”.

The patient who stayed in hospital and her parents regretted the fact that she had to eat her meals alone in her room. They emphasised the harmful aspect of this loneliness around mealtimes imposed by health recommendations.

## Discussion

The aim of this study was to explore the experience of anorexia nervosa patients and their parents following a premature return home as a result of the exceptional conditions imposed by the global pandemic.

Our study identified seven superordinate themes in the interviews with the children and their parents. Two of those could be broken down further into different subthemes. The history of hospitalisation due to anorexia nervosa did not impact these themes. All the patients and parents viewed the premature discharge from hospital positively (except one father), despite displaying initial ambivalence. Children with a history of hospitalisation were less worried about discharge. This was not the case for parents for whom the history of hospital admissions made no difference.

It is very likely that these themes and subthemes would also have emerged on discharge at the end of the weight contract, given the specific characteristics of anorexia nervosa.

- *Concerns*: Fears about relapse are therefore legitimate as they frequently arise. In our study, one of the five patients discharged prematurely had to be readmitted after 4 months because she lost weight. This is consistent with international literature which shows relapse rates ranging from 31% [20] to 41% [21] or even 52% in some studies [22], with variability depending essentially on the definition of relapse used in the study [22]. These relapses mainly occur in the first few months following hospital discharge [20, 21, 23].
- *Preparation*: Patients with anorexia nervosa have difficulty in coping with uncertainty [24, 25]. They also try to avoid being uncertain as much as possible. This intolerance to uncertainty may explain their feelings surrounding lack of preparation and those of their parents.
- *Loss of landmarks and hospital security*: Moreover, most anorexia nervosa patients have greater mental rigidity than control subjects [26, 27]. This may explain the difficulties experienced by both children and parents when faced with the loss of hospital landmarks which they described as “reassuring”. As a remark, some patients commented on a greater ability to adapt, which could be linked to the cognitive remediation therapy sessions focusing on mental rigidity [28] available to them in hospital.
- *Relational aspects*: The intra-familial repercussions of anorexia nervosa are also well documented in the literature [29, 30]. Research has recently focused on the adverse effects on siblings [31]. This difficulty was highlighted by one mother in our study.
- *Likelihood of disease progression*—A better understanding of the disease: Hospitalisation also calmed family tensions [32], which could explain the improvement observed in the relationship. However, parents had a more rounded view and a better understanding of the symptoms in our study. This is probably due to the regular medical consultations organised during the hospital stay. Parental involvement and empowerment in managing the feeding of their children improve childhood and adolescent outcomes [33].

- *Likelihood of disease progression*—Motivation: Even if patients were offered to prematurely end their period of hospitalisation, they always have to reach the discharge weight. Otherwise, a rehospitalisation was planned at the end of lockdown. This was an external motivator and contingency contracts are frequently used in clinical practice [34, 35]. Moreover, during their hospitalisation, patients may have improved their motivation to change (internal motivation) through specific cares as motivational interviewing [36]. Indeed, patients with anorexia nervosa are often characterised by high levels of ambivalence and denial of illness [37]. Several studies have shown that motivation was an important predictor of treatment outcome in anorexia nervosa [38, 39]. This concept was mentioned by parents and one patient in our study and it is important to take this into account in the care.
- *Likelihood of disease progression*—Relationship with food: Finally, the fear of gaining weight and the ensuing anxiety are part and parcel of the very definition of anorexia nervosa and one of the DSM-V criteria [1].
- *Gradual return to a “normal” life*—Education: That being said, because of lockdown measures, the children avoided schooling. Exposure to others, which is a source of anxiety for patients suffering from anorexia nervosa, was also limited. The importance attached to how individuals are perceived by others is associated with the disturbance in body image which is a symptom of anorexia nervosa [40, 41].

Moreover, the literature highlights the importance of self-oriented perfectionism in anorexia nervosa [42]. This could be responsible for high anxiety levels in those patients with secondary low self-esteem. Keeping these worries at bay has benefited the patients in our study and raises the question of proposing adjustments to schooling on discharge from hospital in the anorexia nervosa context.

Some themes emerged from the parents’ discourse but were not mentioned by the patients, and vice-versa.

- *Positive aspects of a premature return home*: Only the mothers said that they were reassured and relieved because their daughters would not be contaminated in the hospital. This could be linked to the overprotective characteristics displayed by the mothers of children and adolescents presenting anorexia nervosa [43]. As far as the patients were concerned, they did not know much about the virus because access to the information was limited in the unit.
- *Gradual return to a “normal” life*—Family/Friendship: Only parents mentioned the satisfaction of being at home all day long with their daughters. Higher parental protection in patients with anorexia nervosa is reported in the literature [43–45]. This overprotection was not perceived by the children. In their study, A.M.H. Albinhac et al. demonstrated that anorexia nervosa patients did not perceive overprotection, rejection or any indifference [43]. Otherwise, parents were probably satisfied to be at home all day long because they were then also able to eat with their daughters. We can assume that they find it reassuring to see what their child is eating.
- *Relational aspects*: According to B. Brusset [46], anorexia nervosa constitutes a “self-centred process which constrains the subject”. The definition per se (“someone who is excessively concerned with himself”) explains why only parents mentioned relational aspects in our study.
- *Likelihood of disease progression*—Relationship with food: Only patients expressed a continued food-related fear. Fear of food is a core symptom of anorexia nervosa and is also known to persist following inpatient care, as highlighted in previous studies [47]. The re-establishment of a kind of care contract with their daughter regarding the meals that scared her, shows that parents are partially aware of their daughters’ difficulties even if they do not mention them directly.

It appears that lockdown has triggered an increase in anorexic symptoms in the non-hospitalised adult population [48, 49] and in the paediatric population [50] around the globe.

This was not evident in this study but patients discharged from hospital during lockdown received intensive follow-up.

This study concludes that all patients and parents expressed positive views. This is encouraging because this alternative therapeutic option cannot be applied instead of standard care if patients and their parents do not trust this care strategy. NICE recommendations [13] state that “whether or not the person is medically stable, within 1 month of admission review with them, their parents or carers and the referring team, whether inpatient care should be continued or step down to a less intensive setting.” That reinforces the idea that a shorter hospital stay could be beneficial for some patients.

One of the strengths of this study was the inclusion of the parents, thereby facilitating a hetero-assessment of patients’ experiences that could overcome alexithymia often observed in anorexia nervosa patients [51, 52].

In addition, interviews were conducted by FL in order to avoid desirability bias as patients and their family were usually followed up by ML.

To our knowledge, this study is the only one of its kind in France to adopt a qualitative approach to investigating patient/parent experiences.

However, there are several limitations in this study. Firstly, the sample size was limited. Nevertheless, data saturation may have been reached as no new element emerged in the interviews with the children and parents at the end of analysis. Secondly, no male subject was enrolled in the study as anorexia nervosa is much less common in boys with an estimated sex ratio of 1/10 [16, 53]. If included, their experience might have been different. Thirdly, the authors did not compare patients prior to COVID-19. However, some patients had a history of hospitalisation for anorexia nervosa and this experience was compared to previous hospital stays. Finally, only the restrictive type of anorexia nervosa was represented in the study whereas the binge-eating/purging type is a predictive factor of relapse [21, 54] and of unfavourable disease progression [55]. The results might then have been rather different in this particular population.

## Conclusions

To conclude, this study highlights the positive experience of anorexia nervosa patients and their parents following early discharge from full-time hospitalisation, with a shift to intensive outpatient care and the absence of face-to-face schooling exposing them to other people.

Changes in care would therefore affect outpatient care post-discharge. Such changes could include a reduction in the length of the hospital stay to facilitate a gradual return home with intensive follow-up (at least three times a week) in line with the weight contract, or an adjustment in terms of the gradual return to school in the subject’s own time.

Further longer-term studies involving larger cohorts will be required to explore these possibilities and to corroborate this positive experience several months after discharge. Experimental studies may also validate alternative care options in children presenting anorexia nervosa.

It would also be interesting to explore the patient experience outside this highly specific context and compare findings.

## Data Availability

The datasets generated and analysed during the current study are not publicly available due to privacy and ethical restrictions but are available from the corresponding author on reasonable request.

## Abbreviations

BMI: Body Mass Index
DSM-V: Diagnostic and Statistical Manual of Mental Disorders, 5th Edition
HAS: Haute Autorité de Santé
